# Annexin A5 Promoter Haplotype M2 Is Not a Risk Factor for Recurrent Pregnancy Loss in Northern Europe

**DOI:** 10.1371/journal.pone.0131606

**Published:** 2015-07-02

**Authors:** Liina Nagirnaja, Diana Nõmmemees, Kristiina Rull, Ole B. Christiansen, Henriette S. Nielsen, Maris Laan

**Affiliations:** 1 Human Molecular Genetics Research Group, Institute of Molecular and Cell Biology, University of Tartu, Tartu, Estonia; 2 Department of Obstetrics and Gynaecology, University of Tartu, Tartu, Estonia; 3 The Fertility Clinics, Rigshospitalet, Copenhagen University Hospital, Copenhagen, Denmark; 4 Department of Obstetrics and Gynaecology, Aalborg University Hospital, Aalborg, Denmark; Institut Jacques Monod, FRANCE

## Abstract

**Introduction:**

Annexin A5 is an essential component of placental integrity that may potentially mediate susceptibility to phenotypes of compromised pregnancy. A promoter haplotype termed M2 of the coding gene *ANXA5* has been implicated in various pregnancy complications such as preeclampsia and recurrent pregnancy loss (RPL), however with inconclusive results.

**Study subjects and methods:**

A retrospective case-control study combining resequencing and restriction fragment length polymorphism (RFLP) analysis was undertaken in 313 women with unexplained RPL and 214 fertile women from Estonia and Denmark to estimate the RPL disease risk of the M2 haplotype in Northern Europe. Comparative prevalence of the studied *ANXA5* genetic variants in human populations was estimated based on the 1000 Genomes Project (n = 675, whole-genome sequencing data) and the KORA S3 500K dataset of South German samples (n = 1644, genome-wide genotyping data).

**Results:**

Minor allele frequency of common polymorphisms in *ANXA5* promoter was up to two-fold lower among Estonian RPL subjects than fertile controls. The M2 haplotype was not associated with RPL and a trend for decreased prevalence was observed among RPL patients compared to controls both in Estonia (8.1% vs 15.2%, respectively) and Denmark (9.7% vs 12.6%). The high M2 prevalence in fertile controls was consistent with estimations for European and East Asian populations (9.6%-16.0%).

**Conclusions:**

This study cautions to consider the M2 haplotype as a deterministic factor in early pregnancy success because: i) no RPL disease risk was associated with the haplotype in two clinically well-characterized RPL case-control study samples, ii) high prevalence of the haplotype among fertile controls and world-wide populations is inconsistent with the previously proposed severe impact on early pregnancy success, iii) weak impact of M2 haplotype on the production of ANXA5 protein has been established by others.

## Introduction

Recurrent pregnancy loss (RPL) defined as three or more consecutive early pregnancy losses before gestational week 22 affects 1–3% of fertile couples aiming at child birth [1]. The heterogeneity of RPL disease etiology has been well established including but not limited to inflammatory, anatomical, immune and thrombophilic factors [2,3]. Due to familial inheritance of the disease [4,5], the genetic contribution has been extensively studied, however with very few factors identified as specific to the RPL phenotype or conferring high risk of the disease (reviewed in [6]).

Both acquired and heritable thrombophilia have been considered as one of the risk factors leading to impaired placental blood circulation and placental infarction resulting in adverse pregnancy outcome. A multitude of studies addressing the inherited thrombophilic mutations, including factor V c.1691G>A (Leiden) and prothrombin c.20210G>A polymorphisms as the most common examples, have reported an association with early pregnancy loss, however with highly variable risk estimations and no conclusive evidence for clinical recommendations [7–9]. Association with increased risk have rather tended to be stronger for fetal deaths, such as stillbirths after 20 weeks’ gestation, than for recurrent early pregnancy losses [7,9].

A combination of polymorphisms in the core promoter of *annexin A5* gene (*ANXA5*, located at 4q27) termed as M2 haplotype has been associated with increased risk of recurrent early pregnancy loss in Europe and Asia and thus proposed as a prevalent genetic determinant of RPL at the earliest stages of pregnancy [10–14]. Annexin A5, also termed as the placental anticoagulant protein, is a ubiquitously expressed phospholipid-binding annexin abundantly found in the placenta. Previously, ANXA5 has been reported as a protein that forms a protective antithrombotic shield at the surface of placental syncytiotrophoblasts [15]. Disruption of this layer and reduced levels of annexin A5 protein on the placental villi have been considered a risk factor leading to increased incidence of pre-eclampsia and early pregnancy loss as demonstrated for women with antiphospholipid and anti-annexin A5 antibodies [16–18]. Most recently, ANXA5 has been described as an endogenous membrane repair protein essential for the integrity of a healthy placenta [19].

This replication study aimed to address the polymorphisms and M2 risk haplotype at the *ANXA5* promoter in RPL patients and fertile controls of two Northern European populations, Estonia and Denmark. The study features one of the largest collections of RPL women (n = 313) to date analyzed for the association between *ANXA5* M2 haplotype and RPL. A combination of resequencing, restriction fragment length polymorphism testing and comprehensive analysis of obtained data in comparison with publicly available human whole-genome sequencing and genotyping datasets was employed.

## Materials and Methods

### Ethics statement

The study was approved by the Ethics Review Committee on Human Research of the University of Tartu, Estonia and the Ethics Committee on Human Research of the Capital Region, Denmark. The study was conducted according to the Declaration of Helsinki principles. Written informed consent was obtained from each individual prior to recruitment and collection of blood samples for DNA extraction.

### Study subjects

The current study included female RPL case-control subjects from two Northern European countries, Estonia and Denmark (in total, n = 185 and n = 342, respectively) (Table 1). The study subjects from Estonia were recruited at the Women’s Clinic of Tartu University Hospital and Nova Vita Clinic, Tallinn, Estonia since 2003. The Danish subjects have been recruited at the Danish Recurrent Miscarriage Clinics, Copenhagen and Aalborg, Denmark since 1986. All study participants were of white European descent. Both sample sets have been well characterized and addressed in RPL research previously [5,20–24].

**Table 1:** Characterization of RPL patients and fertile controls from Estonia and Denmark at the time of recruitment.

			Median age, years (range)		No of women		
Study group	Population	No of subjects	At event^a^	At recruitment^b^	With no live births	With live births (births/patient mean ± SD)	No of miscarriages/ patient, mean±SD [range]
*Fertile women* ^*c*^							
All women	Estonia	99	32 (21–43)	34 (23–44)	0	99 (3.5±1.1)	0
	Denmark	115	30 (22–38)	35 (24–45)	0	115 (2.2±0.4)	0
*RPL women* ^*d*^							
All women	Estonia	86	30 (19–41)	31 (21–43)	45	41 (1.5±0.7)	3.8±1.0 [3–7]
	Denmark	227	30 (19–43)	31 (21–43)	119	108 (1.2±0.5)	4.0±1.3 [3–10]
Early miscarriages (<12 gw)^e^	Estonia	77 (89.5%)	30 (19–37)	31 (21–40)	40	37 (1.5±0.7)	3.7±0.9 [3–7]
	Denmark	171 (75.3%)	30 (19–43)	31 (21–43)	95	76 (1.1±0.4)	4.0±1.3 [3–10]
Late miscarriages (12–22 gw)^e^	Estonia	0	n.a.	n.a.	n.a.	n.a.	n.a.
	Denmark	4 (1.8%)	32 (26–38)	33 (27–39)	1	3 (1.0±0.0)	3.0±0.0 [3]
Early+late miscarriages (≤22 gw)^e^	Estonia	9 (10.5%)	31 (20–41)	34 (23–43)	5	4 (1.8±0.5)	4.8±1.4 [3–7]
	Denmark	52 (22.9%)	29 (22–39)	31 (22–40)	23	29 (1.3±0.6)	4.2±1.2 [3–8]

^a^Age at the time of the 3^rd^ miscarriage for RPL patients or at the time of 2^nd^ (Denmark) or 3^rd^ (Estonia) live birth for fertile controls required for recruitment into the study.

^b^Age at the time of recruitment into the study.

^c^Recruitment criteria included at least two (Denmark) or three (Estonia) live births and no previous pregnancy losses at the time of recruitment.

^d^Recruitment criteria included at least three pregnancy losses at the time of recruitment.

^e^Defined based on the fetal development at the time of pregnancy loss.

gw, gestational weeks; n.a., not applicable; SD, standard deviation.

The patient group included 86 women from Estonia (median age 30; range 19–41 years) and 227 women from Denmark (30; 19–43 years) experiencing unexplained RPL (≥3 consecutive pregnancy loss before week 22 of gestation without any identified cause) (Table 1). All recruited RPL female patients and their partners had normal karyotype tested from peripheral blood lymphocyte cultures and known clinical risk factors of RPL were excluded. RPL women had normal menstrual cycles, no uterine anomalies (by hysterosalpingography, hydrosonography or hysteroscopy) and no antiphospholipid syndrome (APS). APS screening had been conducted using lupus anticoagulants and anticardiolipin antibodies for all Estonian and Danish RPL cases; additionally, the evaluation of β2 glycoprotein 1 and serum levels of protein C and free protein S was available for all Estonian patients and for the Danish patients recruited within last 7 years. The carrier status of trombophilia-associated polymorphisms *Factor V Leiden* (p.Arg506Gln, c.6191G>A, rs6025) and *F2*, *prothrombin* (c.20210G>A; rs1799963) has been tested for all Estonian patients and the mutation carriers were excluded in this study. For the Danish RPL patients, this genetic testing has not been part of the routine clinical assessment, as it has remained controversial, whether heritable thrombophilia is a major causative risk factor for RPL. As a limitation of the current study, karyotypes of the conceptuses from miscarried pregnancies in most of the RPL cases were unavailable.

The control group consisted of 99 Estonian (32; 21–43 years) and 115 Danish women (30; 22–38 years) of well-established fertility with no history of pregnancy losses and at least two (Denmark) or three (Estonia) successful pregnancies ending with live birth (Table 1).

### Resequencing and polymorphism detection in Estonia

Genomic region encompassing the M2 polymorphisms within the minimal promoter region of the *ANXA5* gene according to [10] was amplified from 100 ng of genomic DNA of Estonian subjects using primers PCRI_fw (5’-ACCACGCTCTCCTCTCCAG-3’) and PCRI_rev (5’-CCACGCACTATGTTGAGCAC-3’; S1 Fig) and HOT FIREPol DNA Polymerase (Solis Biodyne, Estonia). The obtained PCR product (570 bp) was purified by treating with exonuclease I and shrimp alkaline phosphatase (Thermo Scientific, USA) and subjected to re-sequencing using primer A5_seq (5’-TGGTCGCAGCCCGGGG-3’) and BigDye Terminator kit (Applied Biosystems, USA).

The obtained DNA sequences were analyzed using the Phred, Phrap and Consed package [25], which enables accurate base calling, sequence quality assessment and assembly. Polymorphisms were identified using the PolyPhred program (ver. 6.02; http://droog.gs.washington.edu/polyphred/) and confirmed by manual checking.

### Genotyping of M2 haplotype tagSNP 76G/A by RFLP analysis in Estonia and Denmark

**Fig 1:**
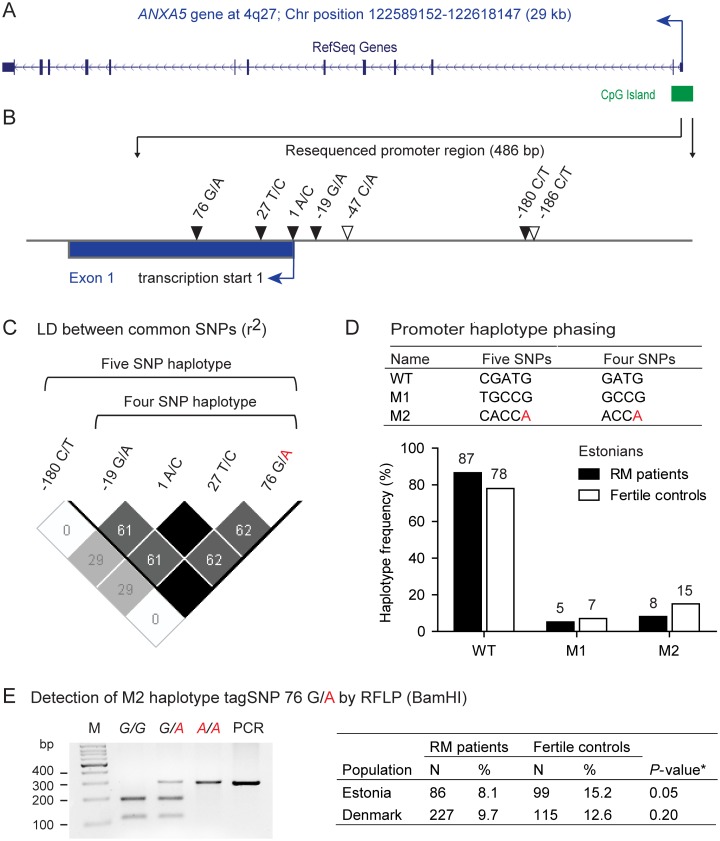
Detection of SNP profile and haplotype distribution within the *ANXA5* promoter region in Estonia and Denmark. (A) Genomic context of the *ANXA5* gene. Chromosomal positions are based on hg19. (B) Location of identified single nucleotide polymorphisms (SNPs) within the resequenced region of *ANXA5* core promoter. SNP positions are given according to the initially reported [10] first transcription start site (arrow) of the non-conserved untranslated first exon (blue box). Common SNPs are denoted with black and rare singleton SNPs with white triangles. SNP nomenclature according to dbSNP database (http://www.ncbi.nlm.nih.gov/SNP/) is the following: -19G/A, rs112782763; 1A/C, rs28717001; 27T/C, rs28651243; 76G/A, rs113588187. In the current human genome assembly (http://www.ensembl.org/), the determined transcription start site is shifted for all annotated *ANXA5* transcripts and only rs113588187 is located within the transcribed region. (C) Linkage disequilibrium (r^2^) between pairs of SNPs within the resequenced region of the *ANXA5* promoter. The order of SNPs is given according to the direction of transcription. Black box indicates complete LD between a pair of SNPs. (D) Distribution of haplotypes identified within the core promoter of Estonian subjects based on haplotype reconstruction analysis with all common SNPs (n = 5) or four common SNPs within the LD block. Haplotype phasing of either five or four SNPs yielded identical results. (E) Detection and observed prevalence of M2 haplotype by Restriction Fragment Length Polymorphism (RFLP) analysis targeting the M2 tag-SNP 76G/A (rs113588187). In case of a *GG* homozygote at position 76, RFLP analysis results in two fragments (188 bp and 106 bp), three fragments are detected in subjects heterozygous for the M2 haplotype (294 bp, 188 bp and 106 bp), whereas one uncut fragment is observed in homozygous carriers of M2 haplotype (294 bp). Minor allele of the 76G/A tag-SNP defining the M2 haplotype is denoted in red. M, molecular weight marker, 100 bp DNA Ladder (Solid Biodyne). PCR, uncut PCR product not subjected to RFLP analysis. N, number of subjects. *Fisher’s exact *P*-value.

A restriction fragment length polymorphism (RFLP) analysis targeting the tagSNP 76G/A of the M2 haplotype (rs113588187; Fig 1) was undertaken to confirm the re-sequencing data of the initial Estonian sampleset with an independent method and to further genotype the Danish RPL case-control samples. The *ANXA5* promoter region containing the tagSNP 76G/A defining the presence (minor allele A) or absence (major allele G) of M2 risk haplotype was amplified from 50–100 ng of genomic DNA using primers PCRII_fw (5’-CGACCACTCACCCAGACTGT-3’) and PCRII_rev (5’-GGAGACCAACTGGGACGA-3’; S1 Fig) and HOT FIREPol DNA Polymerase (Solis Biodyne). The PCR product (294 bp) was subjected to RFLP analysis using BamHI restriction enzyme (Thermo Scientific, USA) that exhibits no sensitivity to DNA methylation and specifically cuts in case of the major allele G at position 76 (Fig 1E; S1 Fig).

### Statistical analysis

The power of the study in both Estonian and Danish RPL case-control sample sets reaches the level that would enable to detect potential association between the common M2 haplotype and RPL. In the Estonian sample set (86 RPL patients, 99 fertile controls), the power of the study is 79.2% based on a two-sided test and considering the following parameters: M2 haplotype frequency 12.4% as estimated for European control samples of 1000 Genomes Project, significance level alpha = 5% and expected OR of 2.85 that represents the average ratio reported by the five studies showing a significant association between *ANXA5* M2 haplotype and RPL [10–14]. The replication sample set from Denmark (227 RPL patients, 115 fertile controls) reaches the power of 94.5% using the same testing parameters.

Conformance to Hardy-Weinberg equilibrium of identified polymorphisms was assessed within study groups using GENEPOP software (version 4.1.4) [26]. The Linkage Disequilibrium (LD) between the polymorphisms was evaluated by calculating r^2^ statistic for pairs of markers using Haploview software (version 4.2) [27].

Haplotypes within study groups were inferred based on unphased genotype data by implementing PHASE software (version 2.1) [28] and using running parameters of 1000 iterations, thinning interval 1, burn-in 100 and—X10 to increase the number of iterations of the final run of the algorithm. Rare singleton SNPs were excluded from the analysis due to unreliable phasing.

The distribution of alleles, genotypes and haplotypes was compared between RPL patients and fertile controls using two-tailed Fisher’s exact test. Association testing was performed using logistic regression analysis with RPL disease defined as the dependent factor, minor allele count or individual genotypes as the independent factors and by adjusting for maternal age at the time of the third pregnancy loss in patients and second (Denmark) or third (Estonia) birth in controls. Common homozygote genotype in the control population was defined as the reference category in the logistic regression analysis of individual genotypes. Bonferroni corrected statistical significance level for *P*-values was estimated for the analysis of individual SNPs (5 tests) α = 0.05/5 = 0.01 and for the analysis of *ANXA5* promoter haplotypes (4 tests) α = 0.05/4 = 0.0125.

### Comparative genotyping dataset of worldwide cohorts

Comparative genotype data for the four SNP positions previously associated with RPL (rs112782763, rs28717001, rs28651243 and rs113588187; Fig 1) [10] were extracted from the publicly accessible 1000 Genomes Project (1000G; http://www.1000genomes.org/) representing genome-wide sequencing dataset of worldwide human population samples. In the current study, we analyzed genotype data of *ANXA5* SNPs for 675 individuals sequenced in the 1000G Project: US residents of Northern and Western European ancestry (CEU, n = 85), Italy (TSI, n = 107), Spain (IBS, n = 107), Great Britain (GBR, n = 91), Finland (FIN, n = 99), Han Chinese from Bejing, China (CHB, n = 97) and Japanese from Tokyo, Japan (JPT, n = 89). In addition, whole genome genotyping dataset of KORA S3 population cohort from Southern Germany [29] and previously reported sequencing dataset from the Netherlands [30] were included. The S3 cohort includes unrelated subjects recruited in 1994–1995 from Augsburg Area (Southern Germany) with a subset of samples (n = 1644, ages 25–69 years) genotyped in the framework of the KORA 500K consortium using Affymetrix 500K oligonucleotide array set (http://epi.helmholtz-muenchen.de/kora-gen/seiten/kora500k_e.php). Genotype distribution of the four *ANXA5* promoter SNPs in the KORA cohort was inferred from the genome-wide genotype data imputed based on the 1000G project dataset.

## Results

### Resequencing *ANXA5* promoter in Estonian RPL patients and fertile controls

Altogether seven single nucleotide variants within the resequenced *ANXA5* promoter region (486 bp) were identified among Estonian RPL patients and fertile controls (n = 86 and n = 99, respectively) (Fig 1A and 1B; S1 Fig and S1 Table). Genotype distribution of all identified variants conformed to Hardy-Weinberg equilibrium in both study groups confirming the accurate sampling and genotyping procedures (S1 Table). Five of the SNPs were found to be common (minor allele frequency, MAF, >5%) and annotated in the database of single nucleotide variants, NCBI dbSNP (http://www.ncbi.nlm.nih.gov/snp/).

Linkage disequilibrium analysis of the five common SNPs identified in Estonian RPL case-control sample revealed a single LD block proximal to the originally reported transcription start site [10] and comprising of four variants previously addressed in association with RPL disease (SNPs rs112782763, originally defined -19G/A; rs28717001, 1A/C; rs28651243, 27T/C; rs113588187, 76G/A; Fig 1B and 1C) [10–12]. The four common variants were occurring with a MAF of 15.2% and 21.7% among Estonian control subjects compared to 8.1% and 13.4% in patients (Table 2; S2 Table). A tendency towards two-fold lower MAF and decreasing odds ratios for genotypes with minor alleles was observed among Estonian RPL subjects, however the association testing by Fisher’s exact test and logistic regression analysis did not reach the Bonferroni adjusted *P*-value (Table 3).

**Table 2:** Prevalence of four common SNPs of the *ANXA5* promoter in this study and in worldwide cohorts.

				Minor allele frequency of SNPs (%)^a^			
Study subjects	Method	Population	N	-19G/A	1A/C	27T/C	76G/A
*This association study*							
RPL patients	Sequencing, RFLP	Estonia	86	8.1	13.4	13.4	**8.1**
	RFLP	Denmark	227	n.d.	n.d.	n.d.	**9.7**
Fertile controls	Sequencing, RFLP	Estonia	99	15.2	21.7	21.7	**15.2**
	RFLP	Denmark	115	n.d.	n.d.	n.d.	**12.6**
*Population-based samples*							
KORA S3	Genotyping	Germany	1644	14.1	22.6	20.6	**12.1**
Hiddink et al. 2012^b^	Sequencing	Netherlands	131	11	20	20	**11**
1000G Project^c^	Sequencing	Europe (CEU)	85	13.5	24.7	23.5	**12.4**
	Sequencing	Japan (JPT)	89	10.1	10.7	11.8	**10.1**
	Sequencing	China (CHB)	97	17.0	16.5	16.5	**17.0**
1000G Project EUR^d^	Sequencing	Finland (FIN)	99	8.1	12.1	12.1	**8.1**
	Sequencing	Great Britain (GBR)	91	11.0	18.1	18.1	**11.0**
	Sequencing	Spain (IBS)	107	10.7	17.8	17.8	**10.7**
	Sequencing	Italy (TSI)	107	13.6	20.1	20.1	**14.0**

^a^Positions relative to the first transcription start site as given in [10]. SNP nomenclature according to dbSNP (http://www.ncbi.nlm.nih.gov/SNP/) and 1000G Project (http://www.1000genomes.org/) databases: -19G/A, rs112782763; 1A/C, rs28717001; 27T/C, rs28651243; 76G/A, rs113588187.

^b^Cohort of healthy controls from Nijmegen, Netherlands [30].

^c^Population-based controls from the dataset of 1000 Genomes Project (Phase 1). Europe is represented by Utah residents (CEPH) with Northern and Western European ancestry (CEU), Japan is represented by Japanese from Tokyo, Japan (JPT) and China is represented by Han Chinese from Bejing, China (CHB).

^d^Extended European population-based controls from the dataset of 1000 Genomes Project (Phase 3). European populations in 1000G Project are Finnish from Finland (FIN), British from England and Scotland (GBR), Iberian populations from Spain (IBS) and Toscani from Italy (TSI).

The tagSNP of M2 haplotype (rs113588187; 76G/A) is indicated in bold.

N, sample size; n.d., not determined.

**Table 3:** Association testing of individual genotypes at four SNP positions in the *ANXA5* promoter with the occurrence of RPL disease.

		No of carriers		Logistic regression^a^	
SNP pos	Genotype	RPL patients	Fertile controls	OR (95% CI)	*P*-value
*Estonian RM case-control sample*					
-19	GG	73	72	1.0	-
	GA	12	24	0.53 (0.24–1.15)	0.117
	AA	1	3	0.32 (0.02–2.64)	0.335
	Fisher’s exact test^b^, *P* = 0.143				
1	AA	64	61	1.0	-
	AC	21	33	0.61 (0.31–1.19)	0.152
	CC	1	5	0.21 (0.01–1.40)	0.166
	Fisher’s exact test^b^, *P* = 0.109				
27	TT	64	61	1.0	-
	TC	21	33	0.61 (0.31–1.19)	0.152
	CC	1	5	0.21 (0.01–1.40)	0.166
	Fisher’s exact test^b^, *P* = 0.109				
76	GG	73	72	1.0	-
	GA	12	24	0.53 (0.24–1.15)	0.117
	AA	1	3	0.32 (0.02–2.64)	0.335
	Fisher’s exact test^b^, *P* = 0.143				
*Danish RM case-control sample*					
76	GG	187	88	1.0	-
	GA	36	25	0.70 (0.39–1.25)	0.220
	AA	4	2	0.94 (0.18–6.88)	0.942
	Fisher’s exact test^b^, *P* = 0.371				

^a^Logistic regression analysis of individual genotypes with the risk to RL; common homozygote genotype in the control population was defined as the reference category and tests were corrected for maternal age at the time of 3rd pregnancy loss for patients and at the time of 2nd (Denmark) or 3rd (Estonia) birth for controls.

^b^Two-tailed Fisher’s exact test of distribution of genotypes in RPL patients versus fertile controls.

OR, odds ratio; CI, confidence interval.

### Promoter haplotype M2 shows a trend for decreased prevalence in Estonian RPL patients compared to fertile controls

In accordance with the high LD in the region, haplotype reconstruction analysis estimated only three promoter haplotypes in Estonia. The number and frequency of inferred haplotypes was identical when analyzing genotype data of either all common SNPs (n = 5) or only SNPs within the identified LD block (n = 4) (Fig 1D).

The three haplotypes detected within the LD block correspond to the *ANXA5* promoter sequences previously studied in RPL case-control samples [10]. The wild type haplotype (denoted as ‘N’) comprised of only major alleles was observed with the highest frequency reaching 86.6% in RPL patients and 78.3% in controls, whereas the lowest prevalence was detected for the M1 haplotype (5.2% and 6.6%, respectively; Fig 1D, Table 4).

**Table 4:** Prevalence of *ANXA5* promoter haplotypes in this and previous studies compared to worldwide cohorts.

	Study subjects					Haplotype frequency (%)^a^					
						N		M1		M2	
Reference	Population	Patients	No of patients	Controls	No of controls	Patients	Controls	Patients	Controls	Patients	Controls
*This association study*											
	Estonia	RPL women	86	Fertile women	99	86.6	78.3	5.2	6.6	8.1	15.2
	Denmark	RPL women	227	Fertile women	115	n.d.	n.d.	n.d.	n.d.	9.7	12.6
*Population-based samples*											
Hiddink et al. 2012 [30]	Netherlands	n.a.	n.a.	Healthy controls^b^	131	n.a.	80^c^	n.a.	8.4^c^	n.a.	11
1000 Genomes^d^	Europe	n.a.	n.a.	Population cohort	85	n.a.	70.6	n.a.	7.6	n.a.	12.4
	Central Japan	n.a.	n.a.	Population cohort	89	n.a.	87.1	n.a.	0	n.a.	9.6
	North China	n.a.	n.a.	Population cohort	97	n.a.	83.0	n.a.	0	n.a.	16.0
*Previous association studies*											
Bogdanova et al. 2007 [10]	West Germany	Pool of cases^e^	70	West German fertile women cohort^f^	500^g^	80.0	82.9	5.7	12.0	14.3	5.1
				North Germany cohort^h^	533^g^		87.9		3.9		8.2
Rogenhofer et al. 2012 [13]	South Germany	RPL couples	30	Fertile women	90	78.3	81.7	5.0	10.0	16.7	8.3
				West German fertile women cohort^f^	500^g^		82.9		12.0		5.1
				North Germany cohort^h^	533^g^		87.9		3.9		8.2
Tüttelmann et al. 2013 [14]	West Germany	RPL women	243	West German fertile women cohort^f^	500^g^	80.9	82.9	7.4	12.0	11.7	5.1
				North Germany cohort^h^	533^g^		87.9		3.9		8.2
	Bulgaria	RPL women	236	Pool of controls^i^	200	84.3	88.0	4.5	4.5	11.2	7.5
Tiscia et al. 2009 [11]	South Italy	RPL women	103	Fertile women	195	78.2	89.0	2.9	3.3	18.9	7.7
Miyamura et al. 2011 [12]	Central Japan	RPL women	243	Fertile women	119	88.9	93.7	<1^j^	<1^j^	10.7	5.5
Hayashi et al. 2013 [36]	Central Japan	RPL women	264	Fertile women	195	88.0	89.0	<1^j^	<1^j^	11.4	9.7
Cao et al. 2013 [31]	East China	RPL women	94	Fertile women	169	n.d.	n.d.	n.d.	n.d.	12.2	14.2

^a^Haplotype frequency is given as reported in the respective study. If not provided, haplotype frequencies have been calculated based on reported haplotype distribution data.

^b^Results of statistical analysis performed for 131 healthy individuals from Nijmegen, Netherlands including 67 male and 64 female subjects.

^c^The frequency of haplotype N is calculated by combining two sub-classes of haplotype N (H1 and H2) determined by Hiddink et al. 2012. The frequency of haplotype M1 was estimated by combining the corresponding sub-classes of M1 haplotype—H4 and H5. The M2 haplotype frequency is equivalent to the haplotype H3 in Hiddink et al. 2012.

^d^Population-based controls from the dataset of 1000 Genomes Project. Europe is represented by Utah residents (CEPH) with Northern and Western European ancestry (CEU), Japan is represented by Japanese from Tokyo, Japan (JPT) and China is represented by Han Chinese from Bejing, China (CHB). Full list of estimated haplotypes provided in S1 Table.

^e^RPL patient group includes women with both pregnancy losses in the first or second trimester (n = 56) and women with stillbirths (n = 14).

^f^Cohort of fertile women from the registry of the Institute of Human Genetics, University of Muenster, West Germany.

^g^Same two cohorts employed in three studies (Bogdanova et al. 2007; Rogenhofer et al. 2012; Tuttelmann et al. 2013).

^h^Female subjects from population-based PopGen biobank at the University Clinic Schleswig-Holstein Kiel, North Germany.

^i^The control group is compiled of 33 fertile women and 167 healthy individuals (102 men and 65 women) from the registry of National Genetics Laboratory.

^j^The haplotype frequency is either <1.0% or not identified in the study.

n.d., not determined; n.a., not applicable.

Interestingly, the M2 haplotype with minor alleles at all positions and previously implicated in RPL was occurring with a frequency of 15.2% among fertile controls compared to only 8.1% in RPL patients, although the potentially reduced disease risk was statistically non-significant (Fisher’s exact test of haplotype carriership, *P* ≥ 0.05; S3 Table). The prevalence of the M2 haplotype determined by resequencing study was subsequently fully confirmed by RFLP analysis specifically targeting the M2 tagSNP (76G/A; rs113588187) that distinguishes M2 based on the haplotype reconstruction data (Fig 1D and 1E).

### Replication study in Danish subjects confirms that M2 haplotype does not confer risk to RPL

A replication study was undertaken to address the M2 frequency in RPL patients and fertile controls of Denmark (n = 227 and n = 115, respectively) employing the M2-specific RFLP analysis. In accordance with the findings obtained for Estonian sample, the observed MAF of the 76G/A polymorphism representing the M2 haplotype was estimated as 9.7% in RPL patients and 12.6% in fertile women from Denmark with a tendency towards decreased disease susceptibility (Table 2; Fig 1E; S2 Table). Altogether, 27.3% of Estonian and 23.4% of Danish control subjects carried the M2 haplotype in an homo- or heterozygous state compared to only 15.2% and 17.7% in RPL patients, respectively, confirming that the haplotype does not increase the risk of having RPL in Northern Europe (S3 Table). The distribution of the 76G/A genotypes in both Danish study groups conformed to Hardy-Weinberg equilibrium (*P* = 0.16 in RPL patients; *P* = 0.88 in controls).

### High world-wide prevalence of *ANXA5* promoter polymorphisms and M2 haplotype is inconsistent with its strong effect on recurrent pregnancy loss

The analysis of human population genetic data confirmed that the *ANXA5* promoter SNPs determining the M2 haplotype represent common genetic variants, which are prevalent across Europe (KORA S3: MAF, 12.1%- 22.6%; 1000G, CEU: 12.4%- 24.7%; across 4 European sub-populations: 8.1%- 20.1%) and Eastern Asia (1000G, JPT: 10.1%- 11.8%; CHB: 16.5%- 17.0%) (Table 2). These allele frequency estimates are comparable to the range observed for Estonian and Danish controls in this study (12.6%- 21.7%) and for healthy individuals from Netherlands as reported by Hiddink et al. (11%- 20%) [30] (Table 2). Consistently, reconstruction of the *ANXA5* promoter haplotypes from the sequencing data revealed similar haplotype distribution and notable prevalence of M2 haplotype in the European-derived CEU sample (haplotype N: 70.6%, M1: 7.6%, M2: 12.4%) as was detected in the Estonian controls (N: 78.3%, M1: 6.6%, M2: 15.2%) (Table 4; S4 Table). Asian populations lacked the M1 haplotype but the M2 frequency was also estimated as 10% or higher. The consistently high prevalence of the M2 haplotype reported for fertile controls and in general European and East Asian population does not support the scenario, whereby the M2 haplotype exhibits a strong effect on the risk of recurrent pregnancy loss.

## Discussion

The *ANXA5* promoter haplotype M2 has previously been associated with an increased risk of RPL in Germans, Bulgarians, Italians and Japanese [10–14]. This replication study aimed to address the contribution of *ANXA5* M2 haplotype in the occurrence of RPL disease in two Northern European populations, Estonia and Denmark. The combination of two genotyping techniques, resequencing and RFLP analysis, followed by comprehensive data analysis and incorporation of comparative *in silico* datasets of European and Asian population-based samples revealed high world-wide prevalence of M2 haplotype and decreased or similar occurrence among the women with RPL.

The *ANXA5* M2 haplotype was detected at lower prevalence in Estonian RPL patients compared to fertile women (8.1% vs. 15.2%, respectively) and similar tendency was observed for Danish study subjects in the replication analysis (9.7% in patients, 12.6% in controls) (Table 4). Comparable allele frequency distribution of the M2 tagSNP 76G/A has previously been reported for Chinese women with MAF 12.2% in RPL patients and 14.2% in fertile controls [31]. The M2 haplotype prevalence determined for the controls in this study and in the report by Cao et al. is concordant with the estimations for the respective population-based samples (1000G project: CEU, Europeans 12.4%; CHB, Chinese, 16.0%) and with the frequency established for healthy cohort subjects from Nijmegen, Netherlands (11%) [30] (Table 4). A recent study reporting the M2 haplotype frequency as high as 23.6% in the Malays (South-East Asia) is supportive to the high world-wide prevalence of this variant [32]. Notably, in some previous genetic association studies [10–14], the reported frequency of the M2 haplotype for the control women was lower (5.1%- 8.3%) than the population frequency estimates based on the 1000G project (9.6%- 16.0%) (Table 4). Subsequently, genetic association testing with the RPL patients who exhibited M2 prevalence (10.7%- 18.9%) similar to the general population identified an apparently increased disease risk associated with the haplotype with odds ratio ranging from 1.5 to 5.2. Likewise, inconsistent results have been reported for the M2 haplotype in pregnancy-related venous-thromboembolism [33–35].

The observed deviation from the previously reported association between the M2 haplotype and RPL may arise from various aspects related to differences in addressed population, experimental setup or study design. For example, differing selection criteria of RPL patients and fertile controls are a common source of inconclusive results as often observed in genetic association studies of the heterogeneous RPL disease [6]. Concordantly, selection criteria vary between the studies addressing the *ANXA5* gene both for RPL patients (differing number and timing of pregnancy losses but also pooled collection of various pregnancy complications) and fertile controls (differing number of live births and pooling with cohort samples) (Table 4).

It should be noted that significant deviation from Hardy-Weinberg equilibrium (*P-*values <0.05) has been reported for control groups incorporated in three out of seven published association studies [10,13–14]. An excess of M2 homozygotes was shown in these controls inconsistent with the dominant mode of inheritance proposed for the involved polymorphisms [36]. The departure from Hardy-Weinberg equilibrium may potentially indicate bias in sampling and/or genotyping procedures frequently overlooked in genetic association studies [37–38]. Biased genotyping of the studied promoter polymorphisms could be introduced by the complex nature of this genomic region located within a CpG island that has a very high GC-content (approximately 75%) (Fig 1A). Furthermore, the tagSNP of M2 haplotype 76G/A is overlapping a CpG site (cg08715877; UCSC genome browser, http://genome-euro.ucsc.edu/index.html) that may potentially affect the experimental procedures and PCR amplification. In this study, the sequencing results were confirmed by an independent well-optimized PCR and RFLP to decrease chances of biased amplification and genotyping results.

The observed heterogeneity in *ANXA5* association studies may arise due to a currently unclear biological impact of the M2 haplotype. Up to 2.7-fold decreased transcription efficiency has been shown for the M2 haplotype in the HeLa cells *in vitro*, however the observed effect remains to be confirmed in the placental cells [10]. Furthermore, the strongly reduced levels of *ANXA5* mRNA are only mildly reflected at protein level indicating a redundancy in annexin A5 metabolism [18,30,39–40]. For example in placental samples of pre-eclampsia, the *ANXA5* mRNA expression level was reduced to 1%, while the protein level remained at 65% of the average normal expression [18].

It has also been shown that alternative genetic variants in the promoter region of *ANXA5* may rather modify the production of the protein. Sub-classes of N and M1 haplotypes and polymorphism rs62319820 (-180C/T; S1 Table) upstream of the ‘classical’ four M2 SNPs exhibit a significant impact on the plasma level of ANXA5 protein in healthy subjects from Netherlands [30]. Considering the location of polymorphisms defining the M2 haplotype within the CpG island (Fig 1A), it is also plausible that other means of variation (e.g. DNA methylation) could potentially modulate the susceptibility to RPL disease. Furthermore, colocalization of annexin A5 with oocyte-specific form of DNA methyltransferase 1 in mouse preimplantation embryos has been observed [41].

Additional level of uncertainty is introduced by the undetermined origin (placental or maternal) of annexin A5 protein at the surface of the human syncytiotrophoblast. Equal contribution of maternal and paternal alleles has been suggested by a pilot study targeting M2 in RPL couples [13], whereas murine *ANXA5* knockout model has shown the crucial role of only maternal ANXA5 production in pregnancy maintenance [42]. Most recently, the continuous presence of placental ANXA5 on the surface of syncytiotrophoblasts has been questioned and an alternative function as an endogenous cell membrane repair protein promoting rapid membrane resealing specifically at membrane tears has been proposed for ANXA5 [19,43]. Thus it currently remains unclear, whether the maternal, fetal or both genetic factors should be assessed when estimating the disease risk of the *ANXA5* gene.

In summary, this study reports high world-wide prevalence of the *ANXA5* promoter haplotype M2 inconsistent with the previously proposed major effect on early pregnancy success. The current genetic association study detected no increased risk of RPL among the M2 carriers in Estonia and Denmark. In order to unequivocally assess the contribution of the M2 haplotype in pregnancy-related diseases, further studies are warranted using other approaches, such as randomized observational trials or conclusive functional experiments to elucidate the effect of annexing A5 variants on gestational metabolism.

## Supporting Information

S1 Table Polymorphisms identified by re-sequencing the promoter region of *ANXA5* gene among the Estonian RPL patients and fertile controls. (DOCX)

S2 Table Association testing of minor alleles at four SNP positions in the *ANXA5* promoter with the occurrence of RPL disease. (DOCX)

S3 Table Prevalence and distribution of *ANXA5* promoter haplotypes among the fertile controls and RPL patients in Estonia and Denmark. (DOCX)

S4 Table All haplotypes identified in the promoter region of *ANXA5* among Estonian RPL cases and fertile controls of this study and among the population samples from the dataset of the 1000 Genomes Project. (DOCX)

S1 Fig Annotated DNA sequence of the genomic region of the *ANXA5* promoter analyzed in this study. (PDF)
