# Supplementary material for: Annexin A5 Promoter Haplotype M2 Is Not a Risk Factor for Recurrent Pregnancy Loss in Northern Europe
Source: PLoS One. 2015 Jul 2;10(7):e0131606. doi: 10.1371/journal.pone.0131606 (PMC4489905; doi:10.1371/journal.pone.0131606)
Supplement: S1 Table — (DOCX) [file pone.0131606.s001.docx]

**Table S1**. **Polymorphisms identified by re-sequencing the promoter region of *ANXA5* gene among the Estonian RPL patients and fertile controls.**

|  | Major/minor allele |  | MAF (%) | | HWE, *P*-value | |
| --- | --- | --- | --- | --- | --- | --- |
| Position^a^ |  | SNP ID^b^ | RPL patients | Fertile controls | RPL patients | Fertile controls |
| -186 | C / T | n.r. | S | - | n.a. | n.a. |
| -180 | C / T | rs62319820 | 5.2 | 6.6 | 1.00 | 0.34 |
| -47 | C / A | n.r. | S | - | n.a. | n.a. |
| -19 | G / A | rs112782763 | 8.1 | 15.2 | 0.53 | 0.57 |
| 1 | A / C | rs28717001 | 13.4 | 21.7 | 1.00 | 0.84 |
| 27 | T / C | rs28651243 | 13.4 | 21.7 | 1.00 | 0.84 |
| 76 | G / A | rs113588187 | 8.1 | 15.2 | 0.53 | 0.57 |

^a^Position relative to the first transcription start site as given in the first genetic association study of *ANXA5* in RPL by Bogdanova et al. 2009, Hum Mol Genet 16: 573-578.

^b^Based on NCBI dbSNP (<http://www.ncbi.nlm.nih.gov/snp>)

MAF, minor allele frequency; HWE, Hardy-Weinberg equilibrium; n.r., not reported; S, singleton heterozygous SNP observed in a single individual, n.a., not applicable.
