# Supplementary material for: Annexin A5 Promoter Haplotype M2 Is Not a Risk Factor for Recurrent Pregnancy Loss in Northern Europe
Source: PLoS One. 2015 Jul 2;10(7):e0131606. doi: 10.1371/journal.pone.0131606 (PMC4489905; doi:10.1371/journal.pone.0131606)
Supplement: S2 Table — (DOCX) [file pone.0131606.s002.docx]

**Table S2. Association testing of minor alleles at four SNP positions in the *ANXA5* promoter with the occurrence of RPL disease.**

|  |  | Minor allele frequency (%) | | Fisher’s exact test^a^ | Logistic regression analysis^b^ | |
| --- | --- | --- | --- | --- | --- | --- |
| Population | SNP pos | RPL patients | Fertile controls | *P*-value | OR (95% CI) | *P*-value |
| Estonia | -19 | 8.1 | 15.2 | 0.052 | 0.54 (0.27- 1.04) | 0.074 |
|  | 1 | 13.4 | 21.7 | 0.041 | 0.57 (0.31- 1.0) | 0.054 |
|  | 27 | 13.4 | 21.7 | 0.041 | 0.57 (0.31- 1.0) | 0.054 |
|  | 76 | 8.1 | 15.2 | 0.052 | 0.54 (0.27- 1.04) | 0.074 |
| Denmark | 76 | 9.7 | 12.6 | 0.241 | 0.77 (0.48-1.27) | 0.301 |

^a^Two-tailed Fisher’s exact test for the distribution of minor alleles in RPL patients versus fertile controls.

^b^Logistic regression analysis for the distribution of minor alleles in RPL patients versus fertile controls, corrected for maternal age at the time of 3rd pregnancy loss for patients and at the time of 2nd (Denmark) or 3rd (Estonia) birth for controls.

Statistical singnificant level was considered *P* <0.01, taking into account the adjustment for testing of five SNPs.

OR, odds ratio; CI, confidence interval.
