# Supplementary material for: Annexin A5 Promoter Haplotype M2 Is Not a Risk Factor for Recurrent Pregnancy Loss in Northern Europe
Source: PLoS One. 2015 Jul 2;10(7):e0131606. doi: 10.1371/journal.pone.0131606 (PMC4489905; doi:10.1371/journal.pone.0131606)
Supplement: S3 Table — (DOCX) [file pone.0131606.s003.docx]

**Table S3**. **Prevalence and** **distribution of *ANXA5* promoter haplotypes among the fertile controls and RPL patients in Estonia and Denmark.**

|  |  | Controls^a^ |  | Cases^b^ |  |  |  |
| --- | --- | --- | --- | --- | --- | --- | --- |
| Population | Haplotypes | No of carriers | Freq (%) | No of carriers | Freq (%) | Haplotype distribution, *P*-value^c^ | Haplotype carriership, *P*-value^d^ |
| *Carriers of haplotype N^e^* | | |  |  |  |  |  |
| Estonia | Homozygous | 61 | 61.6 | 64 | 74.4 | 0.11 | 0.22 |
|  | Heterozygous | 33 | 33.3 | 21 | 24.4 |  |  |
|  | No N | 5 | 5.1 | 1 | 1.2 |  |  |
| *Carriers of haplotype M1^e^* | |  |  |  |  |  |  |
| Estonia | Homozygous | 1 | 1.0 | 0 | 0 | 1.0 | 0.82 |
|  | Heterozygous | 11 | 11.1 | 9 | 10.5 |  |  |
|  | No M1 | 87 | 87.9 | 77 | 89.5 |  |  |
| *Carriers of haplotype M2* | |  |  |  |  |  |  |
| Estonia | Homozygous | 3 | 3.0 | 1 | 1.2 | 0.14 | 0.05 |
|  | Heterozygous | 24 | 24.3 | 12 | 14.0 |  |  |
|  | No M2 | 72 | 72.7 | 73 | 84.9 |  |  |
| Denmark | Homozygous | 2 | 1.7 | 4 | 1.8 | 0.37 | 0.20 |
|  | Heterozygous | 25 | 21.7 | 36 | 15.9 |  |  |
|  | No M2 | 88 | 76.5 | 187 | 82.4 |  |  |

^a^Fertile female controls from Estonia (n = 99) or Denmark (n = 115).

^b^RPL women from Estonia (n = 86) or Denmark (n = 227).

^c^Two-tailed Fisher’s exact test for the distribution of N, M1 and M2 haplotypes in RPL patients and controls.

^d^Two-tailed Fisher’s exact test for the carriership (presence or absence) of N, M1 and M2 haplotypes in RPL patients versus controls.

^e^Not determined for for the Danish sampleset.
