# Supplementary material for: Annexin A5 Promoter Haplotype M2 Is Not a Risk Factor for Recurrent Pregnancy Loss in Northern Europe
Source: PLoS One. 2015 Jul 2;10(7):e0131606. doi: 10.1371/journal.pone.0131606 (PMC4489905; doi:10.1371/journal.pone.0131606)
Supplement: S4 Table — (DOCX) [file pone.0131606.s004.docx]

**Table S4. All haplotypes identified in the promoter region of *ANXA5* among Estonian RM cases and fertile controls of this study and among the population samples from the dataset of the 1000 Genomes Project.**

| Source: | This study | |  | | 1000 Genomes Project | | | | | |
| --- | --- | --- | --- | --- | --- | --- | --- | --- | --- | --- |
| Population (no of subjects): | EST cases (n=86) | | EST controls (n=99) | | CEU (n=85) | | JPT (n=89) | | CHB (n=97) | |
| Haplotype^a^ | Chr count | Freq (%) | Chr count | Freq (%) | Chr count | Freq (%) | Chr count | Freq (%) | Chr count | Freq (%) |
| GAT**G** (N) | 149 | 86.6 | 155 | 78.3 | 120 | 70.6 | 155 | 87.1 | 161 | 83.0 |
| GCC**G** (M1) | 9 | 5.2 | 13 | 6.6 | 13 | 7.6 | 0 | 0 | 0 | 0 |
| ACC**A** (M2) | 14 | 8.1 | 30 | 15.2 | 21 | 12.4 | 17 | 9.6 | 31 | 16.0 |
| AAT**G** | 0 | 0 | 0 | 0 | 2 | 1.2 | 0 | 0 | 0 | 0 |
| GAC**G** | 0 | 0 | 0 | 0 | 6 | 3.5 | 3 | 1.7 | 0 | 0 |
| GCT**G** | 0 | 0 | 0 | 0 | 8 | 4.7 | 2 | 1.1 | 0 | 0 |
| AAC**A** | 0 | 0 | 0 | 0 | 0 | 0 | 1 | 0.6 | 1 | 0.5 |
| ACT**A** | 0 | 0 | 0 | 0 | 0 | 0 | 0 | 0 | 1 | 0.5 |

^a^Haplotype is composed of alleles of four SNPs in the given order: -19G/A (rs112782763), 1A/C (rs28717001), 27T/C (rs28651243) and the M2 haplotype tagSNP 76G/A (rs113588187) highlighted in bold. The haplotype nomenclature for the three main haplotypes according to Bogdanova et al. 2009 (Hum Mol Genet 16: 573-578) is given in the brackets. In addition to the three common haplotypes N, M1 and M2, rare promoter sequences were detected in all three cohorts of 1000 Genome Project with the prevalence below 5%. Two out of five rare haplotypes included the minor allele A at the M2 tagSNP position. These haplotypes were only detected in the Asian in Japanese (JPT) and Chinese (CHB) cohorts with the population frequency <1%. None of these haplotypes were identified in European-derived (CEU) samples, confirming the reliability of extrapolating the prevalence of the M2 haplotype in the North-European populations using the genotyping data of the M2 tagSNP 76G/A.

EST, Estonia; CEU, Utah residents (CEPH) with Northern and Western European ancestry; JPT, Japanese from Tokyo, Japan; CHB, Han Chinese from Bejing, China; Chr, chromosome; Freq, frequency.
