# Supplementary material for: Annexin A5 Promoter Haplotype M2 Is Not a Risk Factor for Recurrent Pregnancy Loss in Northern Europe
Source: PLoS One. 2015 Jul 2;10(7):e0131606. doi: 10.1371/journal.pone.0131606 (PMC4489905; doi:10.1371/journal.pone.0131606)
Supplement: S1 Fig — (PDF) [file pone.0131606.s005.pdf]

Figure S1. **Annotated DNA sequence of the genomic region of the *ANXA5* promoter analyzed in this study.** The PCR primers used for amplification and subsequent sequencing in Estonians (PCRI_fw/rev) and for amplification and RFLP analysis in Danish subjects (PCRII_fw/rev) are underlined. Sequencing primer used for sequencing of the PCRI product in Estonians is depicted in bold italic and the start of high quality sequence subjected to polymorphism calling has been indicated with an arrow. The SNP positions are numbered relative to the first transcription start site according to the first genetic association study of *ANXA5* in RM by Bogdanova et al. 2009 [10]. Nucleotide changes at identified SNP positions are given according to the sense strand of the genomic region and are reversed compared to changes given relative to the direction of transcription (*ANXA5* gene is transcribed from the antisense strand), e.g. 76C/T on sense strand corresponds to 76G/A on antisense strand relative to transcription start site (Supporting Table S1). Five of the seven SNPs identified are common and have been annotated in the database of single nucleotide variants, NCBI dbSNP (http://www.ncbi.nlm.nih.gov/snp/): 76C/T, rs113588187; 27A/G, rs28651243; 1T/G, rs28717001; -19C/T, rs112782763 and -180G/A, rs62319820. The restriction site of BamHI enzyme overlapping with the M2 tagSNP 76C/T is indicated with grey background.

PCRI_fw ***A5_seq*** PCRII_fw

CGACCACGCTCTCCTCTCCAGAAGGAGCCCCCT***CCCCGGGCTGCGACCA***C

High quality seq

TCACCCAGACTGTGGGACCCAAGTGCCCCGAAACCCCGGGAGAGCGGCGG

BamHI site

AGAGCCGGGCGACGGTACGCGGGGCGACGCCGAGATGCAGACGCTGAAGG

76C/T

ATC**C**GGCAGCTGCACCTAGACTGATCCAAGCAACGGAAACGCCAGCGGCC

27A/G Transcription start 1 **1T/G** **-19C/T**

CC**A**CCCCGGCCCTGCCCGGCTTGGCCCG**T**CCCACCCCCGCCAGGGCC**C**CA

-47G/T

ACCGCAGGCCGGCCGCGCCAGCCGG**G**CCCGCCCCTGCCCCGGCCTCAGCG

PCRII_rev

CGCCTGCGTGGGTCGCGGCTCGTCCCAGTTGGTCTCCCGCGGCGCGGACT
GTCCCGCTGGTCTCGCGGGCCTGGGTGGGGAGCGGCCGCAGAGCTGCTGG

-180G/A **-186G/A**

AACCAGGA**G**GAGGC**G**CCGCTCCCCCGAGGGCCCCGGAGACCTCTTGTCCT
CGCGCCGCGGGAAGGGCCTGGGGAGCTGTCCAGGGCTCGGGGTTTTGGAG
GTGCTTGAGCCTCCCTGCCCTCTGTGACCGCAGGAACCGTAGCCTCACTT

PCRI_rev

GGGTGCTCAACATAGTGCGTGGGCGTCTTTTTGGAGGCGAGACAAAATCA
